# Semantic feature norms: a cross-method and cross-language comparison

**DOI:** 10.3758/s13428-023-02311-1

**Published:** 2023-12-20

**Authors:** Sasa L. Kivisaari, Annika Hultén, Marijn van Vliet, Tiina Lindh-Knuutila, Riitta Salmelin

**Affiliations:** Department of Neuroscience and Biomedical Engineering, Aalto University, School of Science, PO Box 12200, FI-00076 Espoo, Finland

**Keywords:** Semantic features, Behavioral norms, Text corpora

## Abstract

The ability to assign meaning to perceptual stimuli forms the basis of human behavior and the ability to use language. The meanings of things have primarily been probed using behavioral production norms and corpus-derived statistical methods. However, it is not known to what extent the collection method and the language being probed influence the resulting semantic feature vectors. In this study, we compare behavioral with corpus-based norms, across Finnish and English, using an all-to-all approach. To complete the set of norms required for this study, we present a new set of Finnish behavioral production norms, containing both abstract and concrete concepts. We found that all the norms provide largely similar information about the relationships of concrete objects and allow item-level mapping across norms sets. This validates the use of the corpus-derived norms which are easier to obtain than behavioral norms, which are labor-intensive to collect, for studies that do not depend on subtle differences in meaning between close semantic neighbors.

## Introduction

Concepts serve as handles to meanings in our physical world and allow us to communicate with other people and navigate in a shared semantic space. Most models of semantics assume that the meanings of concepts can be divided into features (e.g., Cree & McRae, 2003; McRae et al., 1997; Rosch, 1975; Taylor et al., 2007). For example, for a concrete concept, a single feature might describe its taste, feel, or function, whereas a set of features would describe a full concept or a class of concepts. Empirically derived semantic representations, which approximate the weighted feature combinations (aka feature vectors) of concepts, are important tools in neuroscience and cognitive science as well as natural language processing (NLP) applications. These semantic representations can be constructed using large-scale behavioral production methods (Devereux et al., 2014; McRae et al., 1997; Vinson & Vigliocco, 2008) and statistical methods which are applied to pre-existing text corpora (e.g., word2vec; Mikolov et al., 2013a & b). In this study, we compare behavioral production norms and norms created with a corpus-based word2vec method in two linguistically distant languages (Finnish and English). We ask whether the resulting semantic representations are comparable across collection methods and languages.

Behavioral production norms are currently considered the gold standard for creating semantic representations empirically (Devereux et al., 2014; Garrard et al., 2001; McRae et al., 2005; Vinson & Vigliocco, 2008), see also (Buchanan et al., 2013). This method was systematically described and developed by McRae and colleagues (McRae et al., 2005) and it provides explicit information about the properties of target concepts. In this method, participants (typically $n>100$) are asked to explicitly produce as many properties as they can think of for a concept. These free-form descriptions are collected and pre-processed, including parsing the freeform responses to meaningful units (e.g., words or phrases), collapsing synonyms and filtering features using production frequency to exclude idiosyncratic features.

The collection of behavioral production norms is highly labor- and time-intensive, which is why only a handful of production norm sets currently exist. While language research is carried out across the world in many different languages, publicly available behavioral production norms are almost exclusively in English (but see also Kremer & Baroni, 2011). This raises the important question of how much effect the choice of language has on such norms, because if the resulting semantic structure is relatively independent of language context, English norms could be used for research in other languages.

Recently, models which utilize large-scale Internet corpora and neural network models (e.g., word2vec; Mikolov et al., 2013a and b), provide a far less labor-intensive alternative to behavioral production norms. These methods are based on the central claim in distributional semantics, i.e., that words with similar meanings tend to occur in similar contexts (Firth, 1957; Harris, 1954). Thus, these NLP-based models reconstruct the linguistic context in which a word of interest occurs and position it in a semantic space. This semantic space can be used to quantify the relationships of words with one another. These methodologies have gained interest because the vector representations are easy and fast to collect and data is available for a very large number of words. Moreover, such vectors can be constructed for a variety of languages as long as large-enough text corpora are available. Therefore, another relevant question is, to what extent corpus-based semantic norms provide comparable information to behavioral production norms.

This study has two aims. The first aim is to establish and report behavioral production norms for Finnish for a set of concrete and abstract concepts (Aalto production norms). The set includes 199 concrete concepts, including living things such as ‘a dog’ and ‘a carrot’ and nonliving things, such as ‘a hammer’ and ‘a car’. Importantly, we also collect behavioral norms for 99 abstract words such as ‘democracy’ and ‘freedom’. The second aim is to examine the extent to which different ways of collecting semantic feature norms contain comparable information. To this aim, we picked three sets of behavioral production norms: (1) the Aalto production norms, (2) CSLB production norms (Devereux et al., 2014) and (3) Vinson and Vigliocco norms (Vinson & Vigliocco, 2008). The behavioral production norms are compared against a corpus-based feature vector set acquired using word2vec in both English (Mikolov et al., 2013a & b) and Finnish (Kanerva & Ginter, 2014). We test whether the different methods produce a comparable similarity structure and also whether the feature vectors of one norm set can be mapped onto those of another to such a degree that individuation of single items is possible.

## Methods

### Aalto production norms

#### Participants

Two-hundred and thirty-three people recruited from mailing lists for university students and social media responded to an online questionnaire in an online survey tool Webpropol (http://w3.webropol.com). A total of 141 of the participants identified themselves as females and 92 as men. The average age of the participants was 25.2 years (SD = 5.7, this information was not available for five participants) and the average years of education was 14.0 years (SD = 2.0). For each concept, we presented the target word (e.g., “OMENA”, English: an apple) and 15 open text fields to which the participants were asked to list the attributes of the presented target word. The participants were first asked to process a set of 30 concepts. After completing this set, the participants had the possibility to process another set of 30 concepts. The participants received either one or two movie tickets depending on the number of words they completed (i.e., 30 or 60, respectively). Each of the 300 target concepts were presented to on average 34.9 (min = 28, max = 56, SD = 6.4) participants.

All participants provided informed consent prior to participating in the study. The study was approved by the Aalto University Ethics committee. Before the experiment, the participants were instructed to list 5–15 qualities (in Finnish "ominaisuus") of words. The participants were advised to think of answers to the following questions: (1) what is it ["mikä se on"]; (2) what is it [partitive tense, "mitä se on"]; (3) what does it do ["mitä se tekee"]; (4) what does it describe ["mitä se kuvaa"]; (5) where does it belong to ["mihin se kuuluu"]; (6) "what is it purpose" ["mikä on sen tarkoitus"] or (7) what is it used for ["mihin sitä käytetään"]. As additional instruction, the participants were asked list properties as if they would explain the meaning of a thing to a child or a person who has never heard the concept before, to avoid associations, to be as brief as possible and indicate how the qualities are connected to each concept if needed. These instructions were given at the beginning of the experiment. The original Finnish instructions apply to our opinion as well to describe concrete and abstract concepts, with the exception of (1) and (7).

#### Selection of items

The authors first selected a set of concepts that were present in two existing norm studies (Devereux et al., 2014; Vinson & Vigliocco, 2008) and discarded words that were not unambiguously translatable (typically homonyms). Additional concrete words, as well as all abstract words, were selected among of most frequent lemmatized words of Finnish Internet corpus (90th percentile of the corpus distribution in version 3 of the Finnish Internet corpus with 1.5 billion tokens in total (Kanerva & Ginter, 2014). We excluded words which were ambiguous, politically or emotionally charged (e.g., ‘ facism’, ‘love’, ‘destruction’), words which indicated a large super-ordinate or subordinate class (e.g., animal, bird), compound-words and proper nouns. This resulted in an initial larger set of 5000 words which were rated in abstractness using a scale from 1 to 5 by two of the authors (S.L.K and A.H). Words that were rated on average < = 1.5 in this scale were classified as concrete. Words whose abstraction-level was rated 4.5 or higher on average were classified as “highly abstract” words. The words which had average abstraction rating of 3 were selected as “medium abstract” words. We performed the final selection of the stimuli such that that the frequency distribution of concrete words and both sets of abstract words matched as closely as possible. This resulted in a set of concrete words (*n* = 200) and two sets of “highly abstract” (*n* = 50) and “medium abstract” words (*n* = 50). One highly abstract word was excluded because it was a duplicate, resulting in 49 highly abstract words in total.

#### Processing of data

The freeform responses of the participants were subjected to a semiautomated preprocessing procedure. The responses were first automatically lemmatized using the Omorfi parser (Pirinen, 2015). The morpheme borders of highly inflected words in Finnish can be ambiguous, and therefore, the Omorfi automatic parser often produces multiple possible lemmatization options. In order to disambiguate between the options, three independent evaluators judged whether the lemmatization was correct and manually corrected the lemmatization when needed. In the rare cases where the three raters were not unanimous, the lemmatization that was selected by two out of the three raters was chosen.

The initial list of parsed features consisted of 8085 lemmas. In order to reduce the sparsity of the data, synonyms (including derivations) were combined; for example descriptions such as small, smallish, little, and miniature would have been considered a single feature. Stop words ("olla" [are], "voida" [can], "usein" [often], "yleensä" [usually], "pitää" [need], "liittyä" [be part of], "esimerkki" [example]) and their inflected forms were excluded from the final set. The feature production frequency was normalized with the document length (range = 28–56) i.e., the number of respondents for each target word. Features that were listed by at least 10% of the raters for one concept were considered. Therefore, the weight of a feature in a representation is quantified by the proportion of participants who listed the feature out of all respondents for that concept.

#### Corpus-based feature norms

The model of semantic space used in the decoding was estimated separately from a 6-billion token Internet-derived text corpus for English (Mikolov, Chen, et al., 2013a & b) and version 4 of the Finnish Internet corpus (Kanerva & Ginter, 2014), (4 billion tokens). In both cases, the semantic space was built using a word2vec skip-gram model with a maximum context of 5 + 5 words (five words before and after the word of interest). The skip-gram model is an efficient method for learning dense vector representations of words from large amounts of unstructured text data. The objective is to find vector representations that are useful for predicting the surrounding words in a sentence given a target word (Mikolov et al., 2013a & b). The code and the English word2vec embeddings are available online at https://code.google.com/archive/p/word2vec. The Finnish word vector data are available online: http://dl.turkunlp.org/finnish-embeddings/finnish_4B_parsebank_skgram.bin.

#### Existing behavioral production norms

We selected two existing behavioral production norms that have sufficient overlap with the Aalto production norms for comparison. The first set, collected by Vinson & Vigliocco, includes 169 nouns depicting concrete objects (animals, fruits, vegetables, tools, body parts, vehicles, clothing) as well as 71 event words and 216 verbs (Vinson & Vigliocco, 2008). The second set, collected by the Centre for Speech Language and the Brain (CSLB), is a production norm set that comprises 638 concepts describing concrete objects (Devereux et al., 2014). Unfortunately, we could not include the McRae norms (McRae et al., 2005) in the statistical analyses due to insufficient overlap with the Aalto production norms. However, the McRae norms can be compared to the other norms using the online visualization tool (see below; see also Devereux et al., 2014 for a systematic comparison of the CSLB and McRae norms).

#### Comparison of different norm sets

We created a cross-referencing of all items in each of the behavioral norm sets (these items are available at https://github.com/AaltoImagingLanguage/Norms/tree/master/data/SuperNormList.xls). This resulted in a list of 1715 words that occurred in at least one of the norms sets. We excluded words that were not nouns as well as words that had multiple meanings. The analyses focused on 98 words that were shared across all five norm sets. We extracted word2vec vectors in Finnish and English for all these words.

### Machine learning analyses

#### Zero-shot decoding

Zero-shot decoding analyses were run on Python 3 (www.python.org) using Anaconda3 distribution (Anaconda, 2020) and the scikit-learn module (Pedregosa et al., 2011). The machine learning models implemented in this study mapped the semantic feature vectors from one set of norms to the semantic feature vectors of another set. The models were trained by using a subset (*n* = 97) of the altogether 98 targets. In the trained model, each dimension in one norm set is associated with a weighted sum of the dimensions in the other set. The training was performed using ridge regression ($\mathcal{l}$ 2-regularized linear regression). The aim of these analyses was to test whether we can establish a statistically significant mapping between the two norms at item-level.

The model was evaluated after the training using leave-one-out classification. The predicted semantic coordinates of the left-out vector were compared with the original (‘true’) coordinates. The classification outcome was determined using cosine distance. We evaluated the level of statistical significance using a permutation test with 1000 iterations, randomly selected subjects and randomly shuffled order of the semantic coordinates across the target objects. We used an alpha-level of 0.0025. This corresponds to Bonferroni-corrected alpha-level of 0.05 (i.e., 0.05(alpha) / 20(number of models) = 0.0025).

#### Self-organizing maps

The self-organizing map (SOM) (Kohonen, 1990) algorithm was used to visualize the ordering of the semantic feature vectors in a two-dimensional space. The SOM is an unsupervised learning algorithm that produces a discrete representation, a so-called map, of high-dimensional input data using competitive learning. Separate maps were trained for Abstract (*n* = 99) and Concrete (*n* = 199) target sets. The SOM analyses were run using SomToolbox for MATLAB (https://github.com/ilarinieminen/SOM-Toolbox). The trained SOM map was further clustered using K-means. The best clustering was selected based on Davies-Bouldin index (Davies & Bouldin, 1979) for K = 1 …10, and multiple runs for each K.

## Results

### Aalto production norms

We report the Aalto production norms for 199 concrete and 99 abstract concepts (https://github.com/AaltoImagingLanguage/SemanticNorms). The average number of features (NOF) for different semantic categories is provided in Table 1. We also list the average numbers of distinctive features (NOdF), i.e., features that occur in no more than two other concepts, as well as a number of shared features (NOsF), i.e., features that occur in three or more concepts. The mean NOF for concrete words are numerically larger compared to two previously reported norm sets (CSLB: Devereux et al., 2014; McRae et al., 2005; 12.2 and 14.4, respectively), but lower than in the Vinson and Vigliocco set (Vinson & Vigliocco, 2008; range 26.9–32.5 for different categories of concrete objects).

**Table 1:** The average number of features (NOF), number of distinctive features (NOdF), and number of shared features (NOsF) for the Aalto production norms

Category/domain	NOF	NOdF	NOsF
Abstract	21.8 (5.2)	4.5 (2.7)	17.3 (4.5)
Living	20.4 (5.2)	4.2 (2.4)	16.3 (3.9)
Nonliving	21.7 (5.5)	4.5 (2.2)	17.2 (4.9)
Nature	21.7 (4.2)	6.4 (2.7)	15.3 (3.1)
Total	21.5 (5.3)	4.5 (2.5)	16.9 (4.5)

Examples of features listed for concrete and abstract concepts are provided in Table 2. As Table 2 illustrates, also for abstract concepts, the produced features appear intuitively meaningful. A vast majority of the features listed for abstract concepts are abstract concepts themselves.

**Table 2:** Examples of the features listed for abstract words (translated from Finnish)

zebra	airplane	an opinion	democracy	freedom	education	history
to live	air	important	country	to want	to get	time
black	person	strong	fair	to get	important	important
white	wing	formation	power	important	to give	fact
savanna	to transport	different	opposite	thing	thing	student
Africa	speed	own	common	own	offer	old
eater	vehicle	is based on	Finnish	society	need	war combine
striped	user	is different from	influence	person	claim	future cause
relative	engine	discussion	make possible	opposite	preparation	school
hoof	to need	is told	opinion	enable	person	describe
mammal	metallic	emotion	right	justice/court	to help	book
four	to move	an effect	political	to decide	school	to write
grass	sky	many	decision	prison	Finnish	story
animal	goods	think	nation	self	knowledge	narration
lion	big	experience	election	to consist of	to acquire	investigate
herd	cockpit	fact	to be together	different	to become educated	teacher
horse	to fly	knowledge	equal	constraint	learning	event
to dwell	traveling	personal	democracy	exploitation	teacher	past
wild	long	connection	the Parliament	responsibility	working life	resource
lookout	ground	dispute	to vote	to do	job	to get bored
kingdom	building	understand	Greek	liberation	trainer	learning

**Fig. 1:**
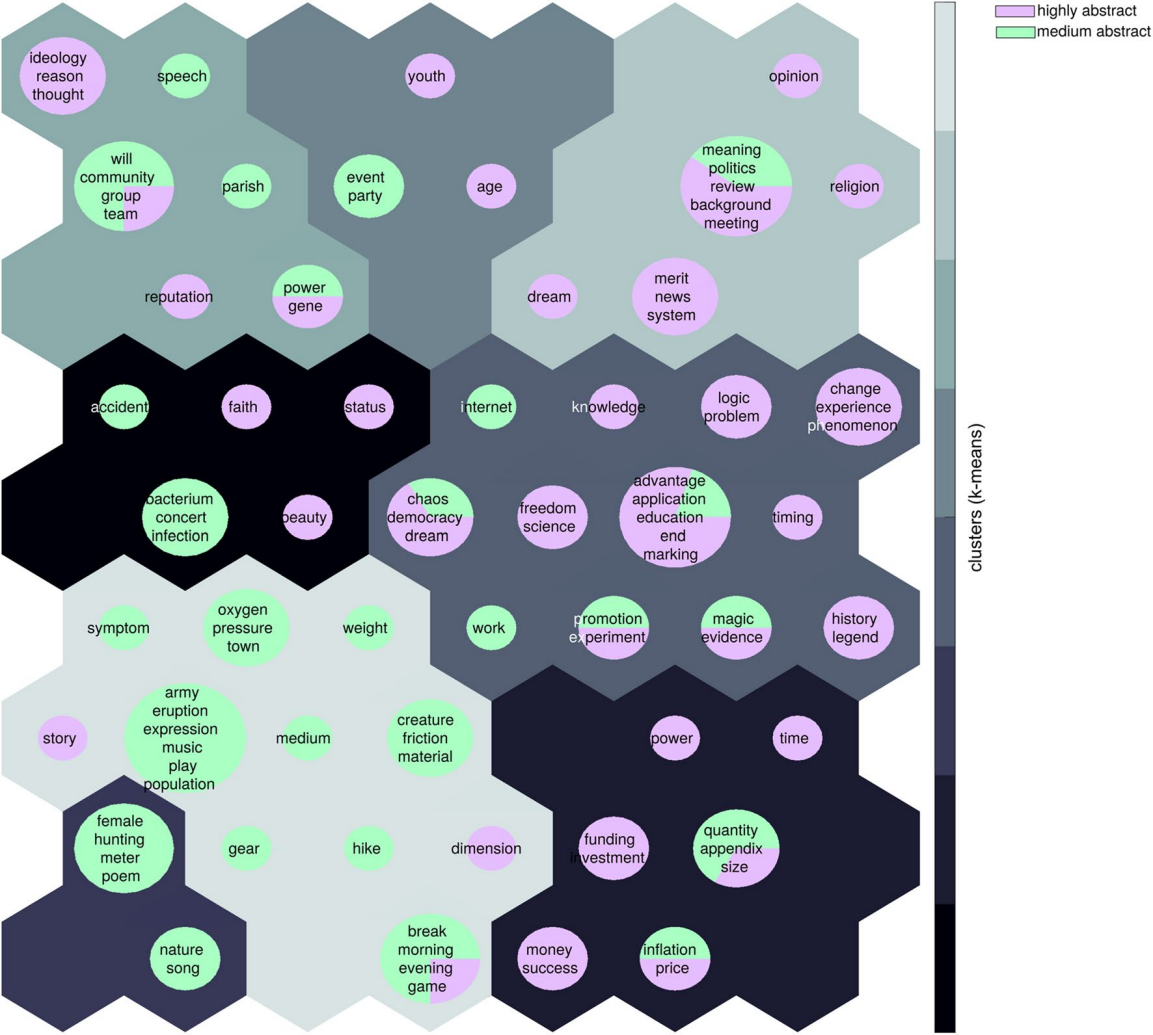
Organization of the abstract target words. Visualization is based on a self-organizing map trained on the feature vectors of the abstract targets in Aalto norms. The self-organizing map is further divided using k-means clustering. The best clustering is selected using Davies–Bouldin index. A toroid map of 60 units was used. In the toroid shape, the units at the opposite edges of the sheet visualized are neighbors

We did not select abstract words based on semantic categories as we did not know whether such categories exist. Instead, we utilized the SOM algorithm and the k-means clustering to visualize the structure of the data in a data-driven manner (Fig. 1). This clustering illustrates that highly abstract words (purple) and medium abstract words (light green) form somewhat distinct categories, with highly abstract words in the right-hand side and medium abstract words in the lower left corner. Beyond that, there are some groupings of words that appears meaningful (e.g., ideology, reason, thought | freedom, science, democracy | funding, investment), but no clear thematic division. This analysis suggests that behavioral production norms tap at least some aspect of the meaning of abstract words as some thematic groupings occur.

### Comparison of different norms

The norm sets compared in the study are summarized in Table 3. The production norm sets have sparse representations, given that each semantic feature represents one dimension. Word2vec produces dense vector representations of a concept where single dimensions are not interpretable.

**Table 3:** Summary of different norms sets

Data	n(concepts)	n(features)	Sparsity	Language	Word types
*Production*					
CSLB	638	2725	Sparse	English (UK)	Object nouns
Aalto	300	1644	Sparse	Finnish	Object and abstract nouns
Vinson & Vigliocco	456	1029	Sparse	English (US)	Object and event nouns and verbs
*Corpus*					
Word2vec (Eng)	> 6B	300	Dense	English (mix)	All types
Word2vec (Fin)	> 2B	300	Dense	Finnish	All types

**Fig. 2:**
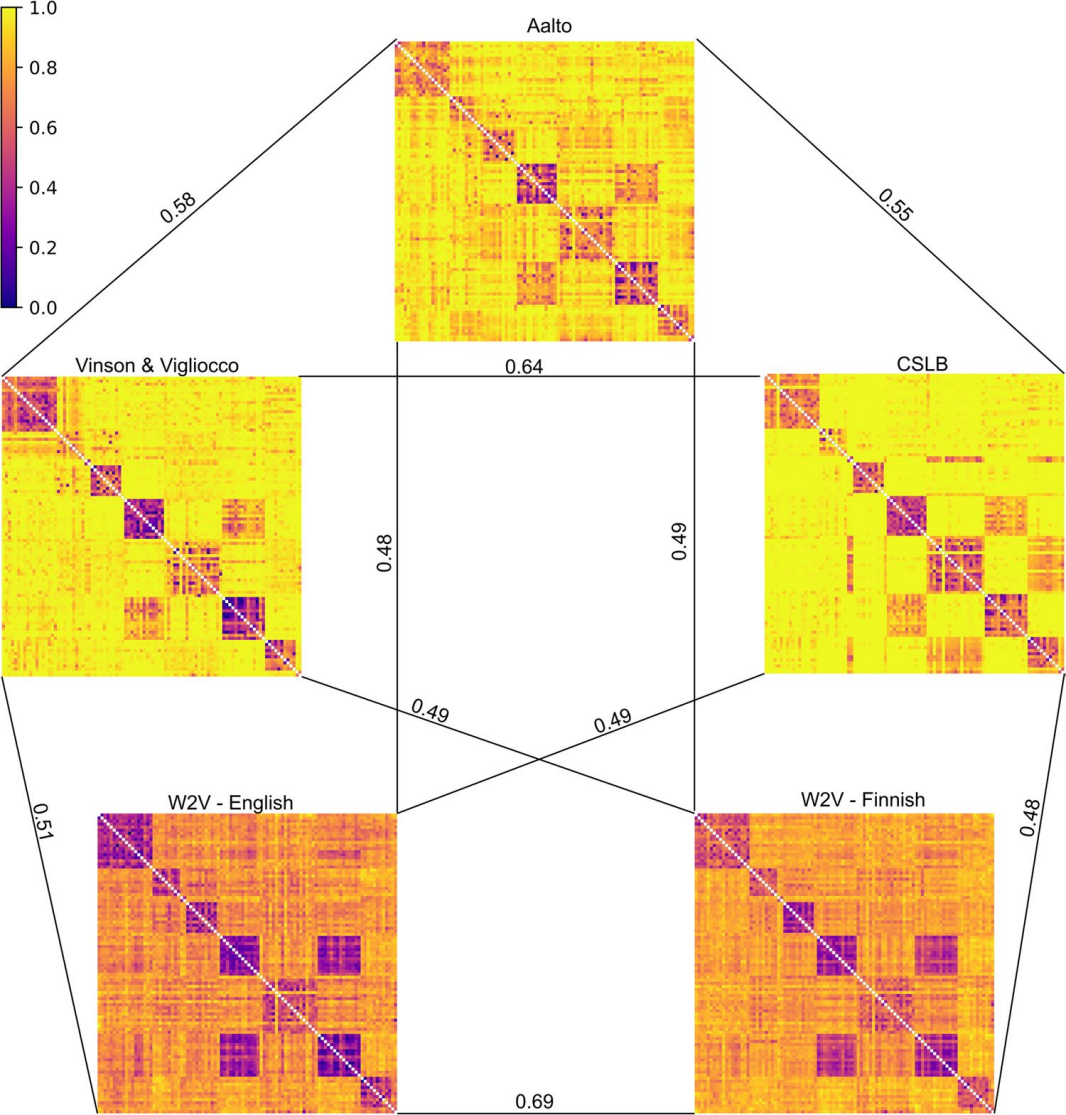
Dissimilarity matrices (cosine distance) of the 98 stimuli shared across the five data sets. The pairwise Spearman rank correlations are indicated in the figure

We calculated Spearman rank correlations across all pairs of upper triangular dissimilarity matrices for all pairs of norms (all *p*’s < 0.001). All correlations were statistically significant after Bonferroni correction. Numerically highest correlations were found for the word2vec norms in English and Finnish as well as the pair of English language based production norms (CSLB vs. Vinson & Vigliocco). The pairwise correlations and the respective dissimilarity matrices based on cosine distances are illustrated in Fig. 2.

Comparisons on abstract words were performed between the Aalto production norms and the Finnish and English Word2vec data. Each pairwise correlation was statistically significant, with the numerically highest correlation between the Finnish and English Word2vec (Spearman’s rho = 0.47, *p* < 0.001), followed by the correlation between Aalto production norms and Finnish Word2vec (Spearman’s rho = 0.32, *p* < 0.001). The correlation between Aalto production norms and English word2vec was also significant (Spearman’s rho = 0.22, *p* < 0.001).

**Fig. 3:**
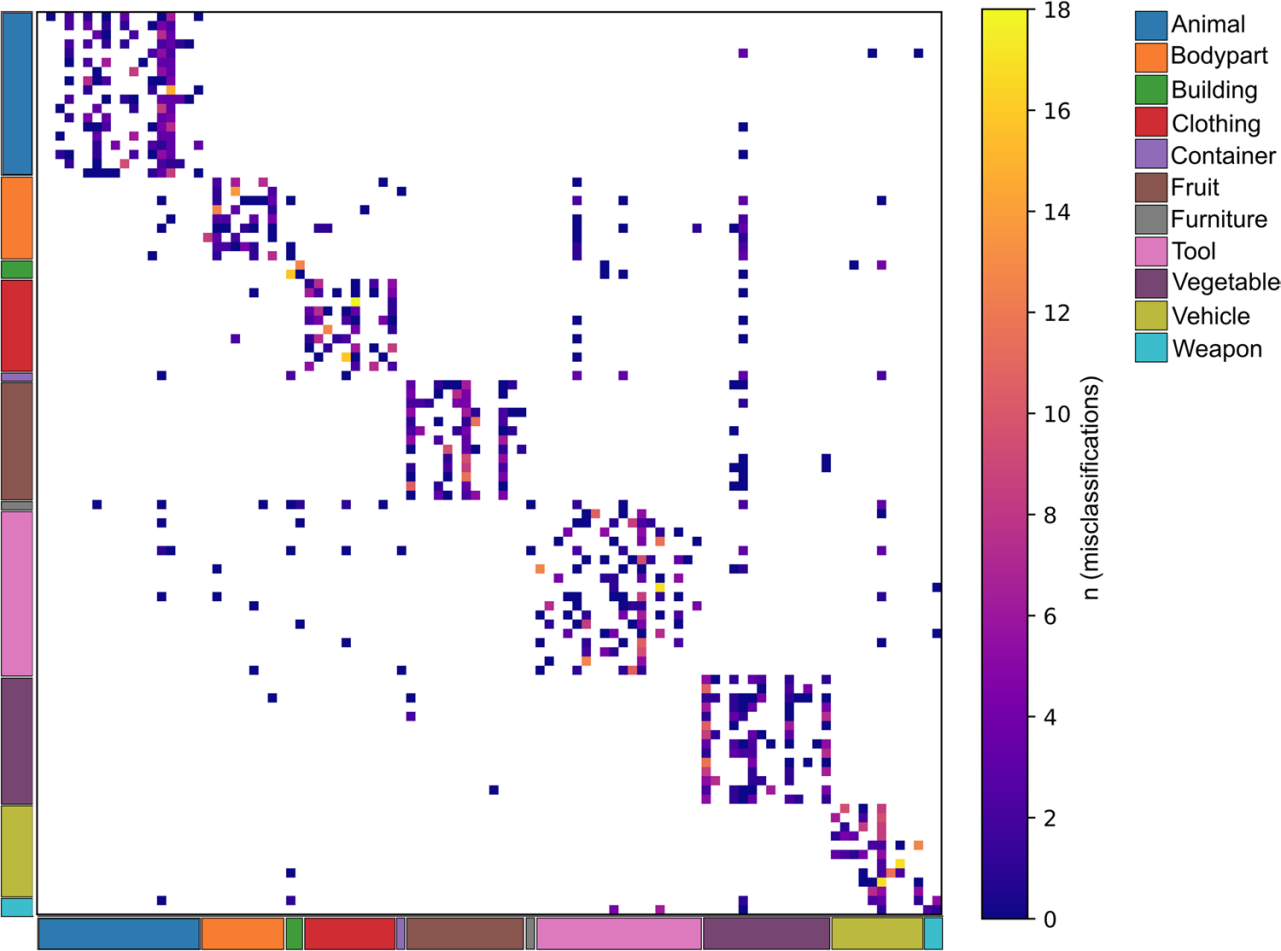
Confusion matrix of the zero-shot learning models. The sum of misclassifications over all the 20 zero-shot learning models is shown. Zero is shown as white to ease interpretation

We performed zero-shot decoding analyses with a leave-one-out classification scheme to examine the extent to which different norm sets can be mapped to one another on item-level. We tested each permutation of the five different norm sets (Aalto, CSLB, Vinson, W2V - English, and W2V - Finnish) which totaled 20 comparisons (see Table 4). For each pair of semantic norms, we performed the zero-shot analysis such that each norm set in turn was used as input data (X) and predicted target data (Y). These analyses demonstrated that in 18 out of 20 cases, the decoding accuracy across norm sets was statistically significant, indicating that it was possible to uniquely identify a single item out of the pool of 98 items beyond chance level. The only models where item-level prediction did not reach statistical significance were those that used CSLB and Vinson & Vigliocco norms as input data and English Word2vec as output data. In these two cases, item-level prediction exceeded chance-level when Word2Vec was used as input data and CSLB and Vinson & Vigliocco norms as output data. All other models predicted concepts at item-level significantly above chance-level based on a permutation test (i.e., 1.0%; Table 4). The confusion matrix across all 20 models illustrates how the misclassifications tended to be such that the predicted concept was in the same semantic category (Fig. 3).

**Table 4:** Zero-shot decoding results. The overall classification accuracy of the zero-shot decoding models using leave-one-out classification. In the leave-one-out classification, the chance-level classification accuracy at the item-level is $1/98\times 100\%\approx 1.0\%$ and at the category-level is $1/11\times 100\%\approx 9.1\%$

>		Item-level		Category-level	
Norm #1	Norm #2	Norm #1 → Norm #2	Norm #2 → Norm #1	Norm #1 → Norm #2	Norm #2 → Norm #1
Aalto	CSLB	11.2	13.3	80.6	83.3
Aalto	Vinson	9.18	18.4	90.7	89.8
Aalto	W2V - English	2.04	5.10	86.7	73.5
Aalto	W2V - Finnish	6.12	8.16	93.9	92.9
CSLB	Vinson	8.16	10.2	85.2	87.8
CSLB	W2V - English	0.00*	8.16	90.8	77.6
CSLB	W2V - Finnish	4.08	8.16	90.9	84.7
Vinson	W2V - English	0.00*	6.12	91.8	80.6
Vinson	W2V - Finnish	3.06	9.18	92.2	90.7
W2V - English	W2V - Finnish	7.14	2.04	92.9	89.8

*Nonsignificant prediction accuracy based on permutation test (*p* > 0.001)

### Visualization of the semantic spaces

An interactive visualization of all five norm sets can be accessed through https://aaltoimaginglanguage.github.io/norms. This visualization uses the embedding projector that was contributed to the TensorBoard project by Luus and colleagues (Luus et al., 2019), allowing the user to interactively explore different types of visualizations of the high dimensional semantic feature vectors. This visualization also includes the McRae norms (McRae et al., 2005).

## Discussion

In this study, we compared behavioral production norms across two linguistically distant languages (Finnish and English) and two collection methods (behavioral production and Word2vec). We found that all semantic feature norms in this study provide a comparable similarity structure across a shared subset of concrete noun words. For abstract words, there also seems to be agreement among the feature norms, albeit weaker than for concrete words. The semantic feature norms, extracted using different methods and in different language contexts, were also sufficiently similar to allow a statistically significant item-level mapping, for the most part. We did not observe systematic differences in the semantic feature vectors between the two languages (Finnish and English).

All norm sets appeared to provide similar information about the semantic category structure of the 98 concrete nouns that were shared across the datasets. However, the correlations appeared to be numerically lower across norm collection methods (behavioral production norms vs. word2vec) compared to within a collection method. These differences are expected, for example, as behavioral production norms are model-driven and often include many quality-assurance steps (e.g., collapsing synonyms, exclusion of taxonomic features or associations, etc.) in order to provide robust and interpretable features (Devereux et al., 2014; Kremer & Baroni, 2011; McRae et al., 2005; Vinson & Vigliocco, 2008), whereas word2vec is fully unsupervised and data-driven. It is inherently biased towards the type of textual material it is based on (e.g., genre, size, linguistic style etc.) which may vary across target concepts (some words occur in Wikipedia, while others are more frequent on public discussion forums). Corpus-based norms are more prone to idiosyncratic associations and polysemy as the data is not collected in an experimental setting, where participants are typically well instructed about the nature of the task. For example, the context of the word "leijona" (English: Lion) in Finnish Internet corpora is influenced by the national ice hockey team, with the same nickname. These factors may explain some of the differences between behavioral production norms and word2vec. In addition to word2vec, there are several different techniques to create corpus-based feature vectors. In our case, all words analyzed were in lemmatized form to match their English counterparts, and word2vec was a straightforward choice in that regard. To cover also inflected word forms, different approaches such as FastText (Bojanowski et al., 2016) or a recent generative model for Finnish nouns based on FastText (Nikolaev et al., 2023) could also be used, as they take into account the sub-word information and generalize also to unseen word forms, beneficial in languages with rich morphology.

We were able to reach significant item-level decoding across almost all feature-norm sets, indicating that each data set carries sufficient information to even be able to distinguish concepts even between individual items. Two exceptions occurred when we used either CSLB norms or Vinson & Vigliocco norms as input data and English Word2vec as output data. This is an ambiguous finding as the reverse was not true (decoding was possible when Word2Vec was used as input data and either CSLB or Vinson & Vigliocco norms as output data). Nonetheless, the nonsignificant result suggests that these two behavioral norm sets may have subtle differences in item-level feature-representations as compared to the English Word2Vec data. Such subtle differences could prove relevant in, for example, neuroimaging studies aiming to map and dissociate neural representations within semantic categories.

Finnish and English have a relatively large linguistic distance which is why norm sets collected in each of these languages makes an interesting study case. Overall, we found that the semantic structure both at item-level and category-level was comparable across Finnish and English norm sets. Based on this, we conclude it is justifiable to use semantic feature norms across languages e.g., in neuroimaging studies. That said, it is important to understand how e.g., syntactic differences in many ways play a role already in the experiment design and data processing options. For example, Finnish has a highly agglutinative morphology which is why it was not feasible to parse the relation (“it has”, “it does”, “it is”) and feature words (e.g., “a shell”, “swim”, “green”) as explicitly as was done by Devereux et al. (Devereux et al., 2014) and McRae (McRae et al., 2005). In Finnish, many aspects of meaning are carried by morphemes which are often not meaningful in isolation. This influenced our decision to reduce the complexity of the vocabulary by lemmatization and other approaches described in "Processing the data". Methodological choices, such as considering each lemma as a single feature, influence the results and particularly parameters such as NOF and NOdF. Such parameters might not be readily comparable across studies but are nonetheless reported, as they may provide relevant information about differences between concepts and how they are processed in the brain (e.g., (Hargreaves et al., 2012; Kivisaari et al., 2012)).

To our understanding, this is the first reported behavioral production set for abstract words (but see also (Vinson & Vigliocco, 2008) for action words). Whereas the concrete words were selected beforehand to be exemplars of distinct semantic categories, no such selection could be done for abstract words. As our SOM analysis indicates, some meaningful clusters emerge (e.g., ideology - reason - thought | funding - investment), but as a whole, the abstract words form a less organized semantic space compared to the concrete words. The feature listings for abstract words in Table 2 indicate that behavioral production methods can be applied to abstract words as well. It should be noted that the behavioral production norms for abstract words have a significant correlation with the same words in the Finnish and English Word2vec data but the correlations are lower than for concrete words. We interpret these data as indicating that behavioral production norms and corpus-based norms may describe some, and potentially different, aspects of abstract words.

It is not possible to determine which norm set provides the most accurate information about the “true” meanings, as each norm set provides merely an approximation of the semantic content of a concept. However, all norms provide corresponding semantic similarity structure and can be mapped to one another at item-level using machine learning. Therefore, we suggest that in many cases, corpus-based methods provide a valid and efficient alternative to behavioral production norms. Depending on the researcher’s needs, the dense semantic feature vectors may also provide a more practical alternative to the sparse feature vectors of behavioral production norms. The results indicate that even across languages, the semantic representations are similar to such a degree that the same norms can be meaningfully employed across different language environments.

## Data Availability

We make available the Finnish set of behavioral production norms and well as a table that can be used for cross-referencing between the different norm sets. These data are available in https://github.com/AaltoImagingLanguage/SemanticNorms. The custom code used in this study is provided online in the same repository. The experiments were not pre-registered.
